# Study on the compaction characteristics of snow on airport runways under vertical load

**DOI:** 10.1371/journal.pone.0332521

**Published:** 2025-09-19

**Authors:** Hanqing Guo, Hao Zhang, Boyuan Ping, Juquan Yang, Yanyu Cui, Qingmiao Ding

**Affiliations:** 1 COMAC Shanghai Aircraft Design & Research Institute, Shanghai, China; 2 State Key Lab. of Civil Aircraft Flight Simulation, Shanghai, China; 3 Civil Aviation University of China, Dongli District, Tianjin, China; Amirkabir University of Technology, IRAN, ISLAMIC REPUBLIC OF

## Abstract

Snow accumulation on airport runways reduces friction, affecting aircraft takeoff and landing performance, as well as operational efficiency and safety. Previous studies have primarily focused on the bearing capacity and mechanical properties of compacted snow. However, there is limited research on the compaction characteristics of naturally loose snow under vertical loads. This study employs the Arbitrary Lagrangian-Eulerian (ALE) method to construct a snow model based on the Modified Drucker Prager Cap (MDPC) Model, analyzing the compaction characteristics of snow under aircraft tires with varying snow thicknesses (5 mm to 50 mm) and vertical loads (250N to 2250N). The results indicate that when the snow thickness is less than the tire tread depth, the impact of vertical load on snow compaction displacement is negligible, maintaining a displacement of 3–5 mm. Conversely, when the snow thickness exceeds the tread depth, the compaction displacement increases with load until reaching a stable state. The compaction rate initially increases and then decreases with different snow thicknesses; beyond the tread depth, the load significantly affects the maximum compaction rate, which reaches 504 mm/s. Additionally, the density changes in the tire-snow contact area are influenced by both snow thickness and vertical load. As thickness and load increase, the duration for which the sidewall density exceeds that of the center also extends, with vertical load having a minimal effect on density transition.

## 1 Introduction

According to definitions established by international organizations, a contaminated runway is characterized by the presence of pollutants such as dry snow, wet snow, slush, compacted snow, frost, ice, or standing water exceeding 3 millimeters in depth [1]. During operations on contaminated runways, the interaction among aircraft tires accumulated snow, and the runway surface creates a complex coupled system involving multiple physical fields.

Accumulated snow or water can significantly reduce the frictional performance of airport runways, increasing rolling resistance and adversely affecting takeoff and landing performance, thereby diminishing aircraft maneuverability and operational safety. Reports indicate that the risk of aircraft veering off the runway in the presence of water or snow is eight times greater than on dry runways [1], with snow-covered runways accounting for over 20% of contamination-related incidents [2]. During takeoff and landing, aircraft speeds can reach up to 200–380 Km/h [3], making the interaction between aircraft tires, snow, and the runway surface particularly sensitive. Under external forces, snow is rapidly compressed, resulting in plastic deformation, which, along with the associated motion and phase changes, alters the tire-snow-runway interaction and subsequently impacts the runway’s frictional performance and rolling resistance [4]. Most existing studies have focused on the bearing capacity and mechanical properties of compacted snow; however, there is a lack of research on the compaction process of loose, naturally occurring dry snow under external loads, limiting the understanding of its dynamic effects during aircraft takeoff and landing.

**Fig 1:**
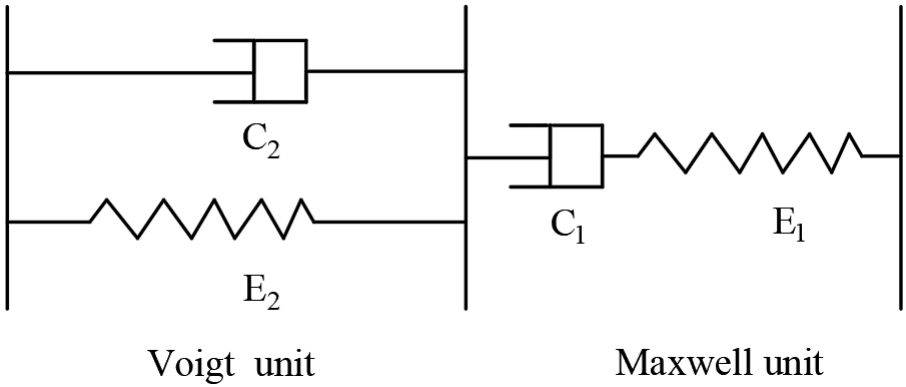
Rheological Model of Snow Compression Characteristics [8].

The testing of snow-related properties is characterized by complexity and variability. The earliest experiments on snow mechanics utilized soil mechanics methods to investigate the mechanical properties of snow [5]. In 1956, Gold [6] conducted uniaxial compression tests and discovered that the compressive strength of snow exhibits considerable variability, influenced by factors such as snow density, crystal size, stress, and temperature, with density being the primary determinant of snow strength characteristics. Landauer [7] employed both constant strain rate and constant load testing methods, revealing that the strain rate of snow is related to its density, crystal size, stress, and temperature; however, the type of snow has a minimal impact on the strain rate. Snow exhibits viscoelastic behavior in response to applied external forces, showing no failure when the external force is sufficiently small. M. de Quervain [8] investigated the viscoelastic properties of snow using laboratory-manufactured snow columns, demonstrating that the mechanical characteristics of snow can be qualitatively represented by a rheological model consisting of a Maxwell unit and a Voigt unit in series, along with a stress relaxation time, τ, as illustrated in Fig 1.

Researchers at Hokkaido University’s Institute of Low Temperature Science [9] conducted quantitative studies on the parameters of the model, indicating that, under certain conditions, the Maxwell simplification model can effectively represent the viscoelastic behavior of snow. This model incorporates stress relaxation time to characterize the stress-strain aging effects of snow, making it suitable for long-term mechanical studies of snow under light loads, though its applicability to the tire-snow interaction remains limited. Between 1957 and 1960, Kinosita [10–11] performed uniaxial tests on natural snow from Hokkaido, finding that the compressive characteristics of snow are influenced by the loading rate. At varying loading rates, snow exhibited both plastic deformation and brittle failure, leading to the identification of a critical range for the transition from plastic to brittle deformation. In 1977, Yong and Fukue [12] conducted confined compression tests on both fresh and aged natural snow, as well as artificial snow produced from ground ice. In 1980, Hidek [13] discovered through uniaxial tensile tests that the deformation patterns and ultimate tensile strength of snow are affected by the strain rate. These findings highlight the numerous factors influencing the characteristics of snow, yet a unified conclusion regarding the extent and patterns of their effects is still lacking, particularly in relation to the changes in snow properties under tire loading.

To overcome the regional and seasonal limitations of snow research, analytical and numerical methods have increasingly been adopted. In 2004, Haehne [14] et al. utilized the Capped Drucker-Prager (CDP) model within the Abaqus finite element software to simulate the deformation-load response of low-density snow (150–250 kg/m³) under high strain rate loading, comparing and calibrating the results with controlled laboratory and field experiments. In 2006, Lee [15] et al. employed a depth-dependent upper-bound indentation model, utilizing the Drucker-Prager material parameters, to investigate the influence of snow depth on tire-snow interaction, performing numerical analyses of tire sinking at various snow depths. In 2009, Lee [16] developed a finite element model using Abaqus to simulate the compression of snow by a rigid plate and an inflated tire, applying the Arbitrary Lagrangian-Eulerian (ALE) algorithm to study the pressure-sinking relationship during tire-snow interaction. The study categorized the sinking trends of tires in snow into three deformation regions: linear elastic, extended hardening plastic, and densification, indicating that the pressure-sinking relationship from plate indentation tests qualitatively resembles that from static tire indentation tests. In 2016, Lee [17] et al. analyzed the interaction between tires and snow using a physical model based on a similar statistical framework for dry snow, resulting in the calibration and validation of a wet snow interaction model. A single test run revealed that dry snow exhibited a lower interface friction coefficient and higher traction compared to wet snow.

In 2018, Zeinab El-Sayegh [18] et al. utilized smooth particle hydrodynamics and hydrodynamic-elastic-plastic materials to model snow, focusing on the interaction between truck tires and snow. They calculated the rolling resistance coefficient of the tires under various working conditions, including vertical load, inflation pressure, tire longitudinal speed, and snow depth, and demonstrated the impact of these conditions on the rolling resistance coefficient of truck tires. In 2020, Cutini [19] et al. investigated the traction performance of winter tires on icy and snowy road surfaces, establishing a method to reveal and evaluate the differences in traction performance of winter industrial tires; however, they did not examine the properties of ice and snow. In 2021, B. Kabore [20] et al. proposed a discrete element method (DEM) model for the inter-particle bonding and collision of snow, simulating snow as a matrix composed of air, meltwater, and ice particles exhibiting sintering effects to describe interactions at the particle size level. This approach aimed at predicting the mechanical behavior of ice particles under varying strain rates using a unified method. Concurrently, B. Peters [21] et al. introduced a particle bond and collision model within the DEM framework to describe interactions of snow at various scales. However, the application of this discrete element method primarily focuses on interactions between snow particles. In 2024, Enzhao Xiao [22] et al. conducted simulation analyses using Abaqus based on experimental data to explore the bearing capacity of compacted loose snow. Yan Qingdong [23] et al. also employed Abaqus to develop a finite element model for rigid wheels interacting with compacted snow, investigating the contact forces between tires and snow; however, neither study explicitly addressed the compaction characteristics of snow.

This study conducts numerical simulations of the compaction characteristics of snow on runways, validated against current research findings:

(1) Analyzing the MDPC constitutive model for snow and the pressure-sinking model.
(2) Establishing a numerical simulation model for the compression of snow by a rigid plate to validate the accuracy of the snow model.
(3) Developing a numerical simulation model for tire-snow interaction on runways to verify the model’s correctness and analyzing the factors influencing the compaction characteristics of snow under varying vertical loads and snow depths.

## 2 Model and validation

### 2.1 Snow constitutive model

**Fig 2:**
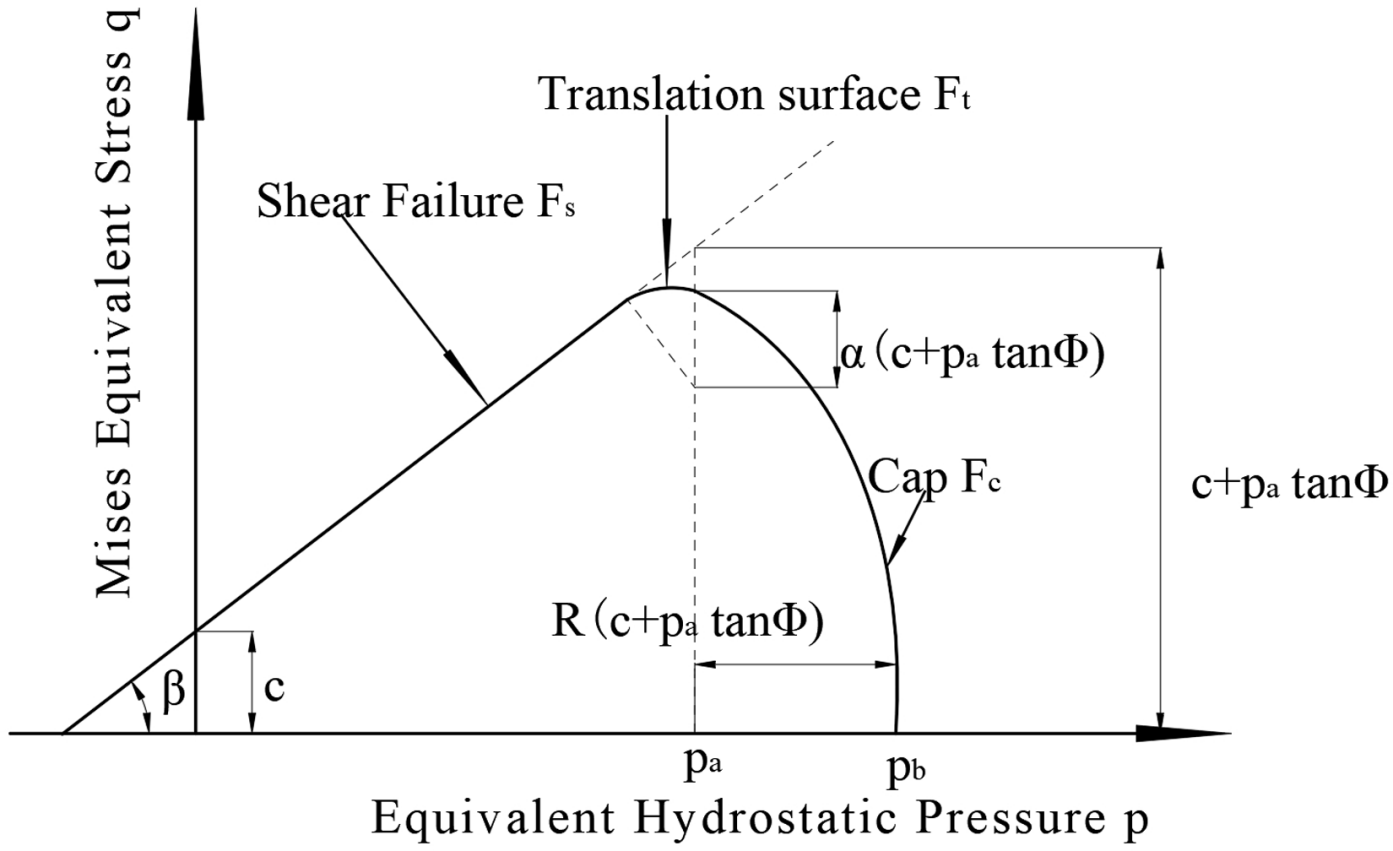
Yield Function of the MDPC Model [24].

In the numerical simulation of tire-snow interaction, commonly used constitutive models for snow incorporate failure yield criteria, such as the Mohr-Coulomb (MC) yield criterion and the Drucker-Prager (DP) yield criterion, with the latter being a generalization of the former. The Modified Drucker-Prager Cap (MDPC) model offers a more detailed representation. Haehnel [14], building on earlier outdoor experiments, studied the snow model and found that the MDPC plastic material model effectively reflects the load-sinking characteristics of snow-soil interaction. Fig 2 illustrates the yield function of the MDPC model [24].

As shown in Fig 2, the model is a linear Drucker-Prager model augmented with a strengthening curve F_c_, referred to as the ‘cap yield surface’. Its primary functions are to provide a non-elastic hardening mechanism during plastic compression and to restrict volumetric deformation in shear failure.

### 2.2 Pressure-sinking model

At a given sinking depth, snow can support a certain level of pressure. Numerous researchers, both domestically and internationally, have studied this phenomenon. The Institute of Low Temperature Science at Hokkaido University [9] conducted experiments to measure the sinking depth of loaded weights in snow, establishing the relationship between pressure and sinking depth for loose snow under specific conditions. Through multiple tests, they derived an expression for the relationship between pressure and sinking depth:


$$P_{m}=\text{4}+\text{1}.\text{70}D+\text{0}.\text{28}D^{\text{2}}$$ (1)

In Equation (1): $P_{m}$ represent the pressure sustained by the snow, (100 Pa); D represent sinking depth of the weight in the snow, (mm).

Yan Qingdong [23] et al determined the Bekker pressure-sinking formula by conducting simulation experiments with circular disks of varying radii, establishing the pressure-sinking relationship under different disk loading conditions:


$$p=\text{2}\left(\frac{\text{30}.\text{81}}{b}+\text{96}.\text{31}\right)z^{\text{0}.\text{44}}$$ (2)

In Equation (2): p represents the normal pressure on the snow, (kPa); z represents the sinking depth of the snow, (mm); b represents the width of the disk, (m).

Yuan Yongli [25] conducted experiments by placing weights of varying masses on snow to measure the distance of free sinking. After multiple trials, a quadratic curve fitting equation was established to describe the relationship between sinking depth and pressure:


$$P=\text{0}.\text{049}D^{\text{2}}+\text{111}.\text{5}D+\text{18}.\text{6}$$ (3)

In Equation (3): P represents the pressure sustained by the snow, (Pa); D represents the sinking depth of the weight in the snow, (mm).

### 2.3 Snow model

#### 2.3.1 Snow material parameters.

To establish the snow constitutive model, it is necessary to determine the parameters through experiments. However, due to significant environmental influences, snow properties can vary widely, and the repeatability of experiments is often poor. Even minor changes in experimental conditions can lead to substantial deviations in results [26]. The snow model employed in this study is the MDPC plastic material model (H200), which represents the plastic deformation of snow. The parameters are shown in Table 1 below. The hardening parameter data for snow is derived from experimental results by Abele [27] from 1975. The parameters were not additionally validated or adjusted for the specific snow conditions used in this study. These parameters have been widely applied and verified and are considered to reliably represent the overall compaction characteristics of snow; therefore, they were directly adopted in this study. However, factors such as snow density, grain size, and internal structure can affect its mechanical behavior and lead to differences in parameters. In this study, the model parameters were not recalibrated, mainly to maintain consistency with previous research and to verify the model’s applicability for general snow compaction simulations. In future work, the parameters could be further optimized through experimental measurements based on different snow layer characteristics to improve the model’s accuracy and adaptability.

**Table 1:** MDPC material parameters for snow.

Parameter	values
Young’s modulus E/MPa	13.79
Poisson’s ratio v	0.3
Drucker-Prager cohesion d/kPa	5
Drucker-Prage angle of friction β/(°)	22.538
Cap eccentricity R	0.2
Initial cap yield surface position $\epsilon_{vol}^{pl}{|}_{0}$	0.001
Transition surface radius α	0
Flow stress ratio K	1
Average snow density ρ/(kg/m³)	200

#### 2.3.2 Validation calculations of the snow model.

**Fig 3:**
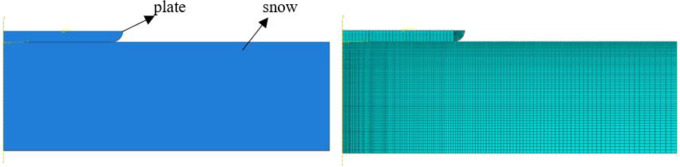
Geometric Model (Left) and Finite Element Mesh Model (Right).

As shown in Fig 3, a finite element model for the plate test was established with a snow thickness of 10 cm and a plate radius of 11 cm. The element type for the plate was set as CAX4R and defined as a rigid body. A constraint was applied to the reference point of the rigid body to restrict displacement in the X-direction, ensuring that the plate does not experience movement in the X-direction during compression, while a downward displacement was applied. The snow model utilized CAX4R elements, with fixed boundary conditions at the bottom to prevent movement. Additionally, the edges of the rigid plate were rounded, and the mesh density was increased to avoid mesh penetration at the contact edges between the plate and the snow, thus preventing stress concentration and computational interruptions.

**Fig 4:**
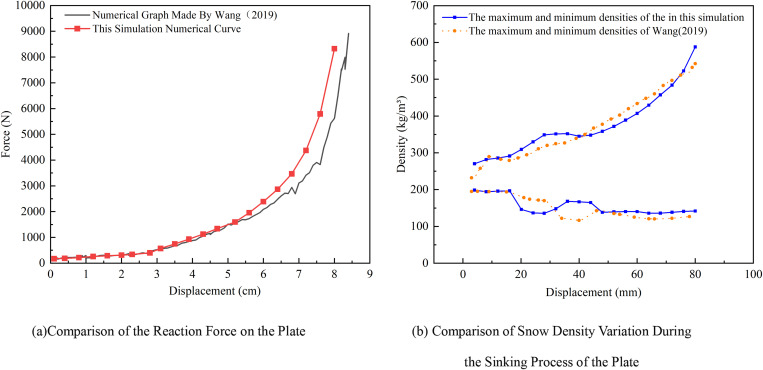
Comparison curves of snow.

Based on the numerical simulation results, the reaction force of the rigid plate is shown in Fig 4a, along with the variation in snow density during the sinking process of the plate in Fig 4b. As the plate gradually presses down, the initial reaction force is relatively small due to the presence of air voids in the snow. However, as the plate continues to compress the snow, the applied force increases rapidly until the snow reaches its final compressed state, at which point the reaction force curve of the plate approaches a vertical alignment. The trends in the reaction force on the plate and the variation in snow density are consistent with the results of Wang Wei [26], with errors remaining within acceptable limits, thus confirming the validity of the constructed snow model.

## 3 Finite element model of aircraft tire-snow runway

### 3.1 Finite element parameter settings

**Fig 5:**
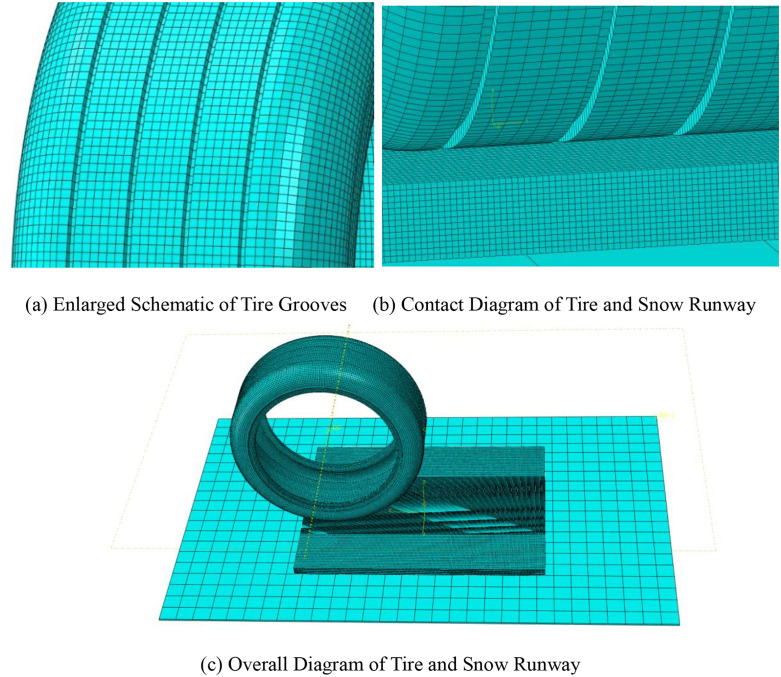
Finite Element Model of Tire and Snow Runway.

A finite element model of the aircraft tire-snow-runway interaction was established (Fig 5). The runway dimensions are 3000 mm in length and 2500 mm in width, and it is defined as a rigid body. The tire model corresponds to the main landing gear tire of the Airbus A320, with a specification of 46 × 17.0 R20, featuring four longitudinal grooves with a depth of 10 mm [28]. The snow model measures 1500 mm in length and 1000 mm in width, utilizing the MDPC model previously validated in the simulations.

The finite element model of the aircraft tire-snow-runway interaction was subjected to mesh generation. In the finite element model of the aircraft tire–snow–pavement system, a refined mesh was applied locally in the tire–snow contact region to accurately capture the mechanical response in this area. Preliminary mesh refinement comparisons in this region indicated that further mesh densification had limited impact on key results such as compaction speed and local density. This suggests that the current mesh resolution is sufficient to represent the main mechanical characteristics of the snow compaction process. Therefore, a multi-scale meshing strategy was adopted for the snow domain to reduce the overall number of elements and improve computational efficiency. Both the tire mesh and the runway mesh were defined using hexahedral elements. The snow model employed linear hexahedral reduced integration elements (C3D8R), with enhanced element hourglass control implemented to prevent convergence issues. Boundary conditions were set with the rigid ground fully fixed, the bottom of the snow completely constrained, and the sidewalls allowing vertical movement freedom. The tire model was released in the vertical direction while retaining degrees of freedom for longitudinal movement and rotation. The Arbitrary Lagrangian-Eulerian (ALE) algorithm was employed, with a friction coefficient of 0.3 set between the tire and the snow [29], this value was adopted based on commonly reported friction coefficients between rubber materials and snow in the literature, without experimental measurement for specific tire materials or snow types. It ensures numerical stability while reasonably capturing the compaction behavior at the tire–snow interface, helping to simplify parameter configuration and focus the simulation on snow compaction under vertical loading. Although the friction coefficient may vary under different snow conditions (e.g., temperature and moisture), preliminary tests suggest that its impact on key outcomes such as compaction velocity and density is relatively limited. Consequently, this finite element model was utilized to analyze the compaction characteristics of snow.

### 3.2 Validation of the finite element model

**Fig 6:**
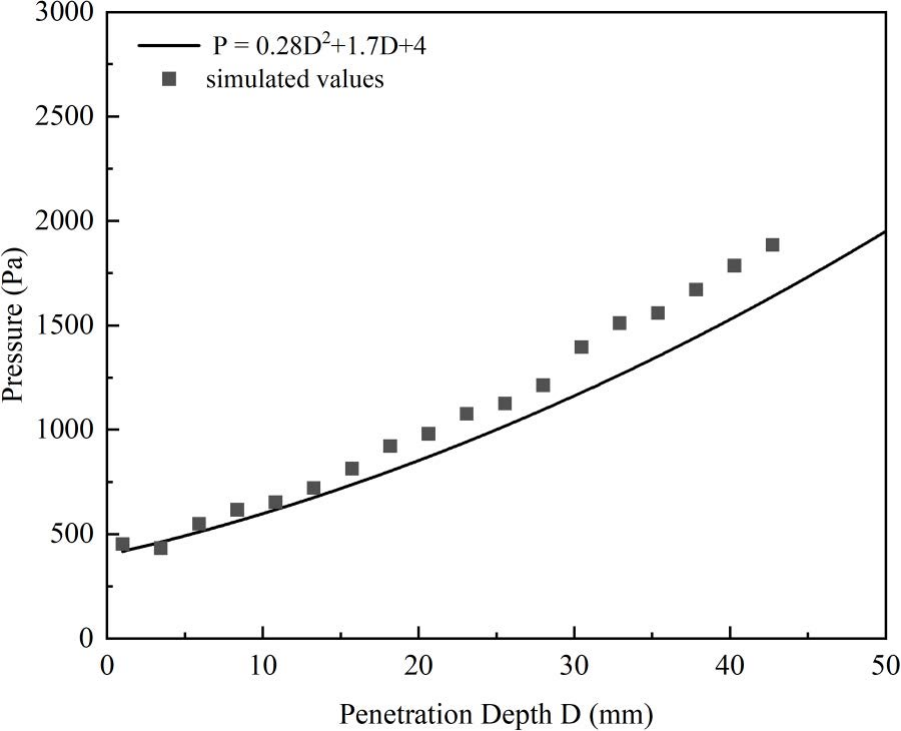
Comparison of simulation results.

Using the pressure-sinking model, simulations were conducted on the established finite element model of the aircraft tire-snow runway to extract the variation in snow pressure at different sinking displacements. The results were compared with the pressure-sinking relationship expressions obtained through experiments at the Institute of Low Temperature Science, Hokkaido University, and the curves are presented in Fig 6.

As seen in the figure, the pressure-sinking data obtained from the simulations closely aligns with the experimental results from Hokkaido University, showing a consistent trend in pressure increase. The average error does not exceed 10%, indicating the validity of the established finite element model. However, a homogeneous and idealized snow model was used in the simulation, and specific conditions such as the snow type, temperature, and humidity in the experiments were not fully replicated. These environmental variables may be one of the reasons for the observed differences. Nevertheless, the model can capture the overall compaction trend well. In the future, incorporating different snow types and environmental factors could further improve the model’s adaptability and accuracy in practical applications.

## 4 Results and discussion

### 4.1 Analysis of snow compaction characteristics under vertical load

In this study, the selected vertical load range of 250–2250 N was primarily determined based on the capacity limitations of commonly used laboratory loading devices and was also informed by typical load settings reported in existing snow compaction experiments. This range ensures a balance between computational accuracy and numerical stability in the simulation. Although the applied loads are lower than the full-scale loads exerted by aircraft tires during takeoff and landing, they are sufficient to capture the compaction trends and localized mechanical responses of snow under varying load conditions. Given that this study focuses on the compaction behavior of the snow layer under static vertical loading within the tire–snow–pavement system, dynamic factors such as shear forces, inertial effects, and tire rolling have not yet been incorporated.

**Fig 7:**
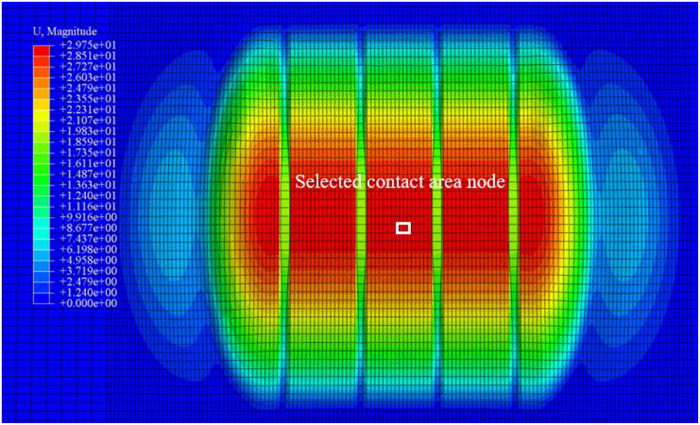
Nodes at the Contact Bottom Position between Snow and Tire.

**Fig 8:**
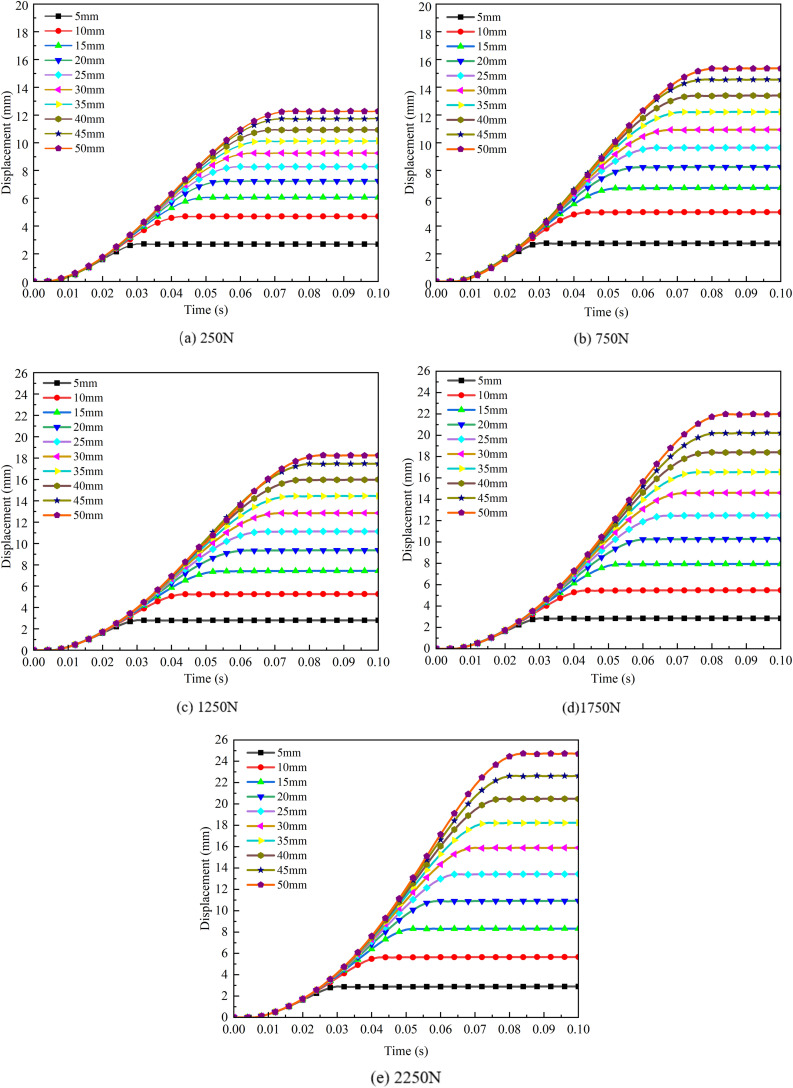
Displacement Changes of Snow Under Different Loads.

An analysis was conducted on the compaction characteristics of snow under vertical loads of 250 N, 750 N, 1250 N, 1750 N, and 2250 N, selecting nodes at the contact point between the snow and the bottom of the tire, as shown in Fig 7. Through simulation calculations, displacement data of the snow was extracted, and a displacement curve for the bottommost nodes at the tire-snow contact interface was plotted, as illustrated in Fig 8.

From the curve, it can be observed that at lower loads (250 N), the displacement change is relatively small, particularly for thinner snow layers with a depth less than the tire groove depth, resulting in an insignificant compaction effect. Under thicker snow (50 mm), the displacement increases, with a maximum displacement of 13 mm, but the overall response remains moderate. However, as the load increases (e.g., 750 N, 1250 N, 1750 N, and 2250 N), the rate of displacement increase becomes significantly pronounced, especially under thicker snow, where the tire produces a notable compaction effect. Thus, larger loads demonstrate a significant capability to compact thick snow, leading to more pronounced displacement growth.

For thinner snow, even under a large load (2250 N), the displacement change remains minimal, with a maximum displacement not exceeding 3 mm. This is because the thin layer of snow lacks sufficient space for further compaction, reaching its deformation limit relatively early. In contrast, for thicker snow, the displacement increases exponentially with the load, indicating that there is more compaction space available for the snow particles under the applied load, resulting in greater compression and displacement.

**Fig 9:**
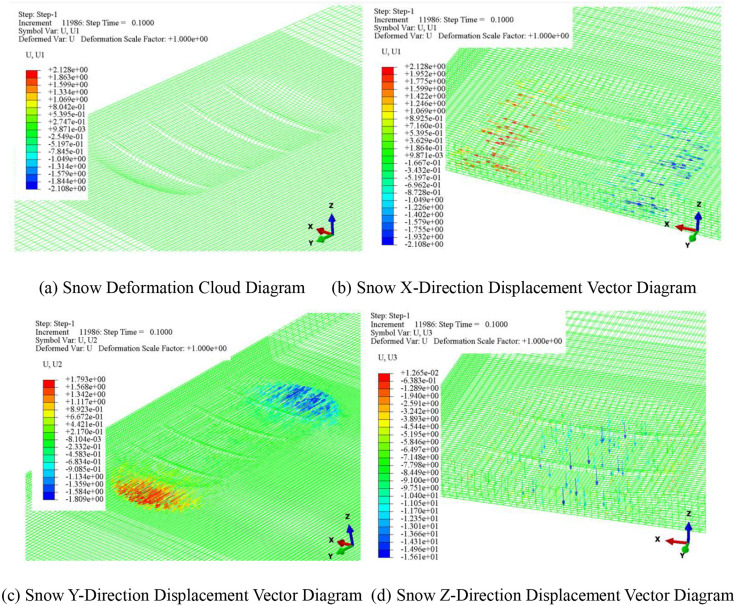
Displacement vector diagram of snow.

Fig 8 shows the selected path diagram, while Fig 9 illustrates the displacement vector diagram of the snow under a vertical load of 750 N and a snow thickness of 50 mm. The compaction displacement of the snow in the tire groove is smaller than that at the tire tread. When the vertical load is applied to the snow, the displacement primarily occurs in the Z direction, which corresponds to the direction of the applied vertical load. According to the displacement vector cloud diagram, the lateral displacement (X direction) of the snow is approximately 2.1 mm, while the longitudinal displacement (Y direction) is about 1.8 mm. The lateral displacement of the snow is greater than the longitudinal displacement, and the snow displacement in the tire’s longitudinal groove is smaller compared to other positions on the tire tread, leading to the appearance of a snow bulge consistent with the shape of the longitudinal tire groove.

### 4.2 Analysis of the Influence of Snow Thickness on Compaction Characteristics

**Fig 10:**
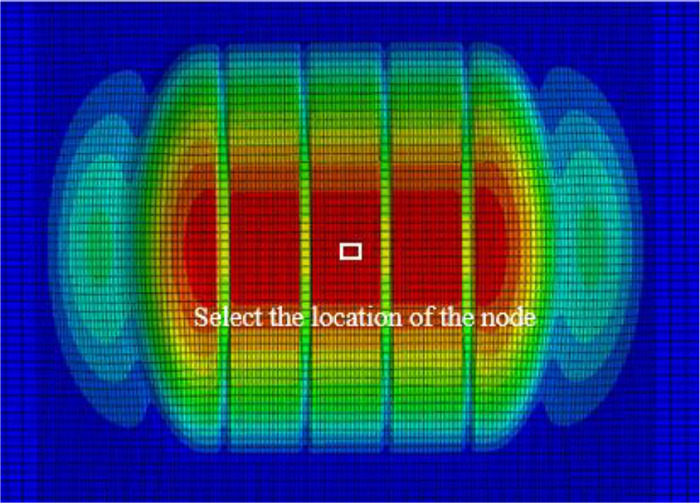
Selected Nodes at the Center of the Tire-Snow Contact Area.

**Fig 11:**
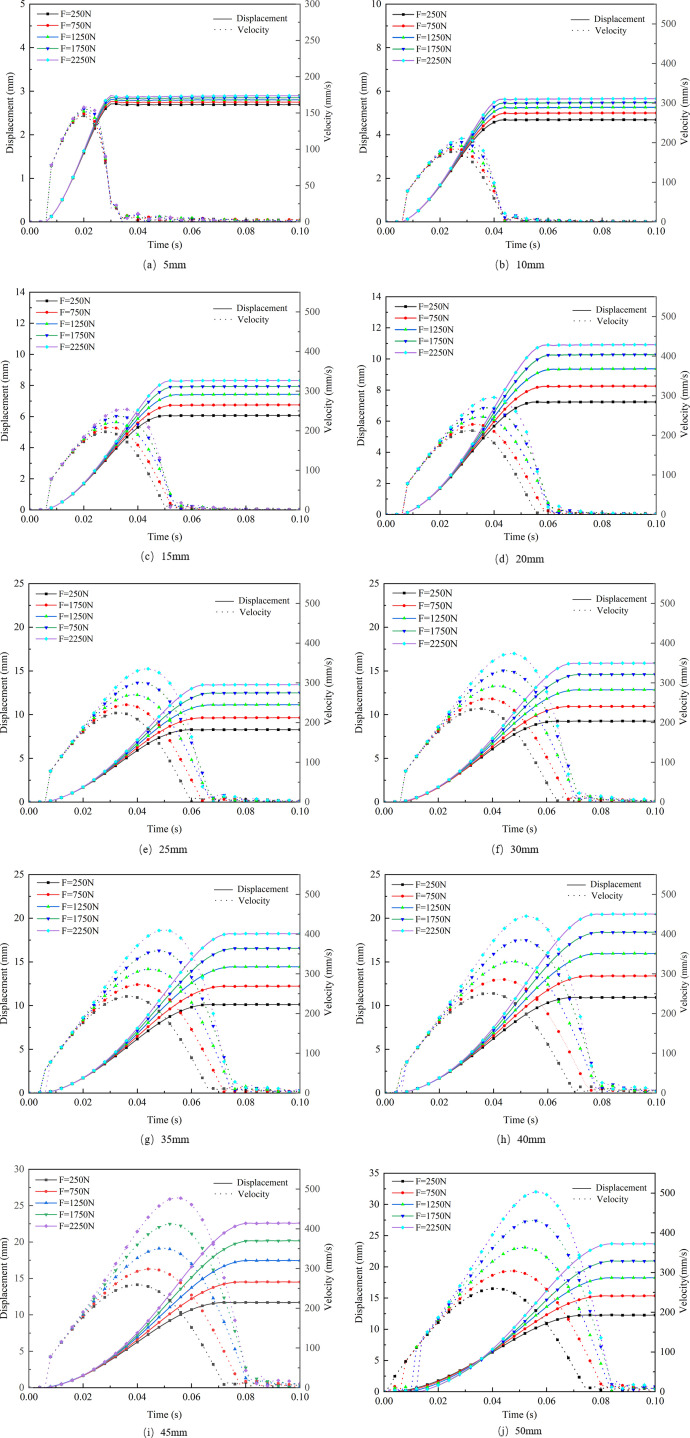
Trends in Displacement and Velocity of Snow at Different Thicknesses.

The interaction between aircraft tires and snow-covered runways is closely related to snow thickness and settlement. As illustrated in Fig 10, nodes at the center of the tire-snow contact area are selected to analyze the compaction effects of snow of varying thicknesses. These nodes represent the changes in displacement and velocity of the snow at the tire’s center. Fig 11 shows the trends in displacement and velocity of the snow under different thicknesses during the tire compression process.

From the curves presented above, it can be observed that under a low load (250 N), the displacement differences for varying snow thicknesses are relatively minor, resulting in a flatter displacement curve. At lower loads, the influence of snow thickness on compaction displacement is less significant than at higher loads. As the load increases (to 1750 N and 2250 N), noticeable differences in displacement curves for different thicknesses of snow emerge, with the compaction displacement of thicker snow being significantly greater than that of thinner snow. Thus, under high loads, snow thickness becomes a critical factor affecting compaction displacement.

Regarding the changes in velocity for snow of different thicknesses, an initial sudden increase in velocity is observed, typically around 0.02 s. This phenomenon occurs because, during the early stages of compaction, the gaps between snow particles are large and the structure is relatively loose, resulting in nearly zero velocity changes and minimal compaction displacement. However, as the load increases, the mutual compression between snow particles intensifies, causing the snow structure to become denser, which leads to a sudden increase in velocity. After reaching the maximum compaction level, the velocity rapidly decreases, entering a stabilization phase.

For thinner snow (5 mm or 10 mm), which is less than the depth of the tire grooves, the initial peak velocity is low and shows minimal variability, reaching a maximum of 210 mm/s and a minimum close to 146 mm/s. The velocity curve quickly stabilizes, indicating a rapid conclusion to the compaction process, with limited space for further compression. In contrast, thicker snow (50 mm) exhibits higher compaction speeds, reaching up to 504 mm/s, and maintains elevated velocity for a longer duration, indicating more available compression space. This phenomenon reflects the visco-plastic response behavior of snow under high strain rates, which is particularly pronounced in loose, low-density snow. At this stage, the internal structure of the snow has not yet formed an effective load-bearing framework, and the bonds between snow grains are rapidly broken, exhibiting typical rate-dependent yielding characteristics. As compaction progresses, the snow becomes increasingly densified, the strain rate decreases, and the compaction speed gradually stabilizes. Overall, the impact of snow thickness on both compaction displacement and velocity is highly significant under high-load conditions, whereas it is relatively minor under low-load conditions.

### 4.3 Analysis of snow compaction characteristics in different regions of the tire

**Fig 12:**
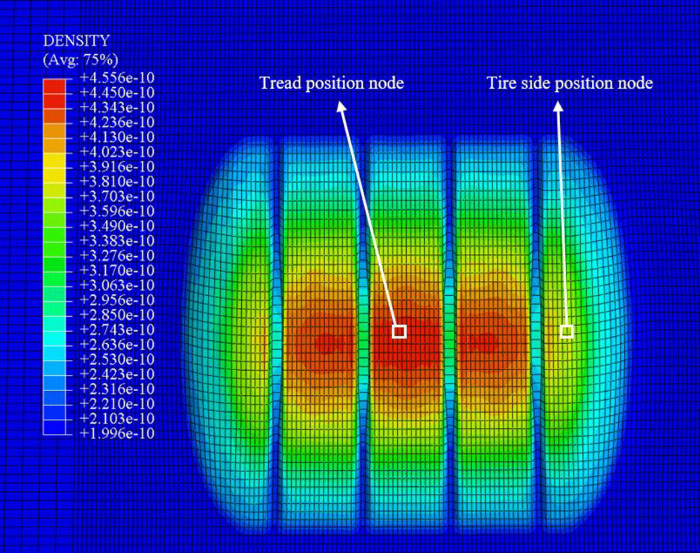
Description of the selected nodes.

**Fig 13:**
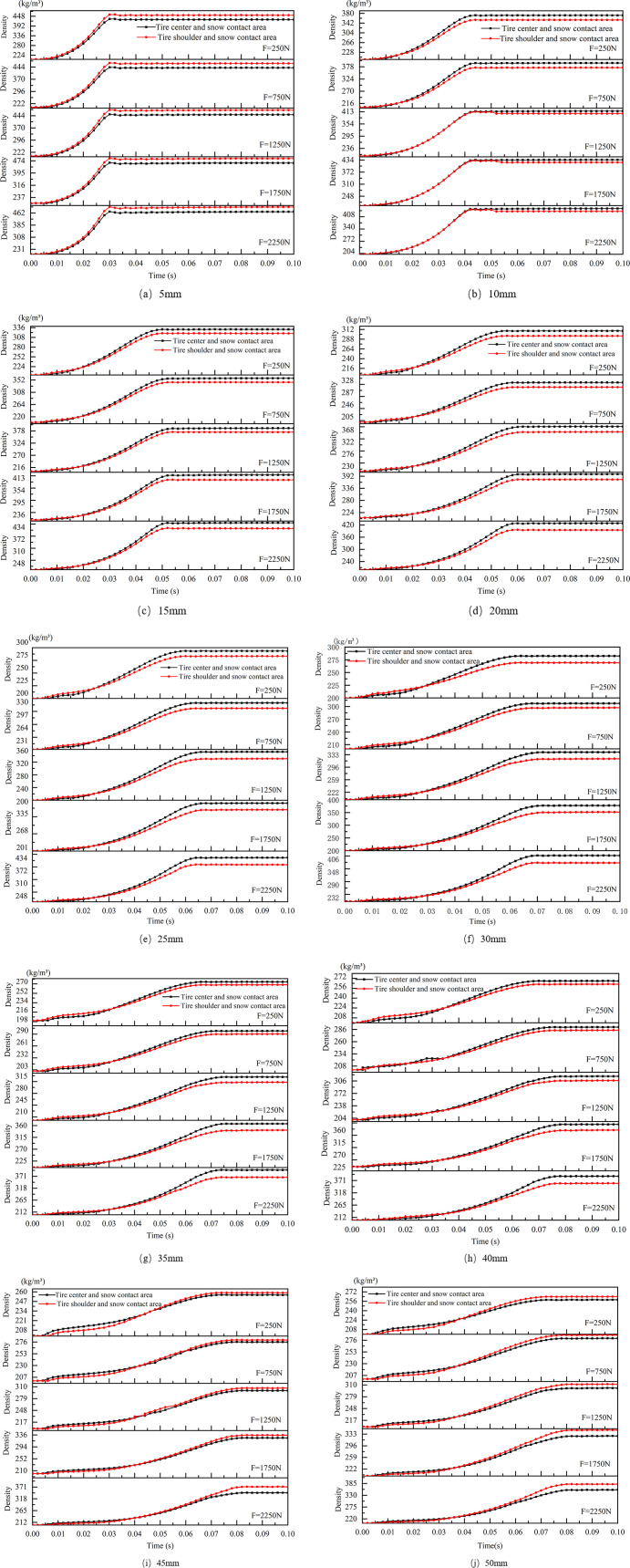
Description of the selected nodes.

Based on the preceding analysis, snow thickness and vertical load exert significant effects on snow compaction. However, variations in the tire’s regions and the state of the snow introduce additional complexities that further influence the contact conditions between the tire and the snow-covered surface. Consequently, two specific regions of the tire—the tread and the shoulder—were selected for detailed analysis. The positions of the selected nodes are depicted in Fig 12, facilitating an investigation into the density variations of snow under different loading conditions. Fig 13 presents the density change curves of snow in both the tread and shoulder regions of the tire across varying snow thicknesses.

Fig 13 the variation curves of snow density in the tire shoulder and tread regions

According to the analysis of the curves, under thinner snow conditions (5 mm and 10 mm), the density curves for the tire shoulder and center regions are closely aligned. Initially, the density increases rapidly, indicating that the snow is compacted quickly, with a maximum final density difference of approximately 20 kg/m³. Notably, under lower loads (250 N), the density curves for the tire shoulder and center overlaps prior to compaction.

In moderate snow thickness conditions, the density in the tire shoulder region is initially higher than that in the tire center, exhibiting a rapid increase in density, while the center density rises more gradually. This discrepancy arises because the contact area between the tire shoulder and the snow is relatively small, yet the shoulder experiences a greater load, leading to more intense compaction. In contrast, the center experiences a more distributed force, resulting in a smoother compaction process. Ultimately, as the tire applies pressure, the final density in the center surpasses that of the shoulder region.

Under larger loads (1750 N and 2250 N), the initial compaction density in the shoulder region is significantly higher than that in the center, with a final density difference of approximately 31 kg/m³. In conditions with greater snow thickness (50 mm), the density in the tire shoulder initially exceeds that in the tire center; however, as compaction progresses, the center density ultimately exceeds the shoulder density, reaching a maximum density difference of 18 kg/m³. Furthermore, as vertical loads increase, the final density difference between the snow in the shoulder and center regions becomes more pronounced, with a maximum difference of 30 kg/m³ observed.

As the thickness of the snow and vertical load increases, the duration during which the density in the tire shoulder exceeds that in the tire center becomes longer. Under thicker snow conditions and larger loads (2250 N and 50 mm thickness), the time during which the shoulder density is higher than the center density is extended. According to the curve variations, this transition occurs at approximately 0.04 seconds under 50 mm of snow thickness, while under thinner snow (such as 10 mm), the transition occurs earlier, around 0.02 seconds, resulting in a time difference of approximately twofold. However, as vertical load increases, the impact on the density transition region becomes less significant.

## 5 Conclusions

This study summarizes and analyzes the mechanical properties of snow and the MDPC constitutive model. A flat plate indentation simulation model is established, and the simulation results are compared with those from current literature to validate the accuracy of the snow model. Based on this snow model, an aircraft tire-snow interface model is developed to investigate the compaction characteristics of snow. The following conclusions are drawn:

(1) The compaction of snow by aircraft tires under vertical load exhibits two distinct characteristics. When the snow thickness is less than the tire groove depth, the vertical load has little effect on the compaction displacement of the snow. Conversely, when the snow thickness exceeds the tire groove depth, the displacement of the snow increases with the vertical load, indicating that greater loads lead to more significant compaction. The displacement primarily occurs in the vertical direction (Z-direction), while the lateral displacement (X-direction) is greater than the longitudinal displacement (Y-direction). The deformation resulting from the compression of the snow corresponds with the shape of the longitudinal tire grooves.
(2) The compaction velocity of snow of varying thickness initially increases linearly, followed by a gradual increase until it reaches a maximum velocity. Subsequently, due to the influence of snow resistance, the velocity decreases slowly until it reaches zero. The displacement of the snow exhibits a gradual increase until it stabilizes, reaching the final compaction level. Furthermore, when the snow thickness is less than the tire groove depth, the variation in the downward velocity of the snow shows little difference, with a minimal velocity difference between maximum and minimum loads, remaining below 100 mm/s. In contrast, when the snow thickness exceeds the tire groove depth, the velocity variation is significantly affected by the load, with differences reaching 200–300 mm/s.
(3) The density variation in different regions of the tire-snow contact area is influenced by snow thickness and vertical load. Under 5 mm of snow, the density in the tire shoulder is greater than that in the center, and the density increases gradually to a maximum, with a final density difference of approximately 20 kg/m³ under varying vertical loads. For thicker snow, the shoulder density initially exceeds that of the tire center; however, as the tire compresses, the center density surpasses the shoulder density. As snow thickness and vertical load increase, the transition time for the tire center density to exceed the shoulder density is approximately 0.02 seconds.
(4) The numerical model of snow compaction proposed in this study not only helps to elucidate the mechanical mechanisms underlying snow–tire interactions but also shows potential for practical engineering applications. The distribution characteristics of compaction speed and local density can be used to characterize the compaction state of runway surfaces, assisting airport operators in evaluating the load-bearing capacity and risk levels of different snow-covered areas. This provides theoretical support for snow removal strategy planning and runway condition assessment.
